# Author Correction: Corrosion-resistant cobalt phosphide electrocatalysts for salinity tolerance hydrogen evolution

**DOI:** 10.1038/s41467-023-44299-4

**Published:** 2023-12-14

**Authors:** Xinwu Xu, Yang Lu, Junqin Shi, Xiaoyu Hao, Zelin Ma, Ke Yang, Tianyi Zhang, Chan Li, Dina Zhang, Xiaolei Huang, Yibo He

**Affiliations:** 1 State Key Laboratory of Solidification Processing, Center of Advanced Lubrication and Seal Materials, School of Materials Science and Engineering, Northwestern Polytechnical University, Xi’an, Shaanxi 710072 P. R. China; 2 Institute of Material and Chemistry, Ganjiang Innovation Academy, Chinese Academy of Sciences, Ganzhou, 341000 China

**Keywords:** Electrocatalysis, Hydrogen storage materials

Correction to: *Nature Communications* 10.1038/s41467-023-43459-w, published online 24 November 2023

The original version of this Article contained an error in Page 4, which incorrectly read *‘the C 1 s spectrum presents one peak around 248.8 eV*.’ The correct version states *‘the C 1 s spectrum presents one peak around 284.8 eV’* in place of ‘the C 1 s spectrum presents one peak around 248.8 eV’. This has been corrected in both the PDF and HTML versions of the Article.

In this article the author’s name *‘Junqin Shi’* was incorrectly written as *‘Junqing Shi’*. The original article has been corrected.

