# Supplementary material for: Corrosion-resistant cobalt phosphide electrocatalysts for salinity tolerance hydrogen evolution
Source: Nat Commun. 2023 Nov 24;14:7708. doi: 10.1038/s41467-023-43459-w (PMC10673868; doi:10.1038/s41467-023-43459-w)
Supplement: Supplementary file 1 — Supplementary Information [file 41467_2023_43459_MOESM1_ESM.pdf]

Supporting Information

Corrosion-resistant Cobalt Phosphide Electrocatalysts for Salinity Tolerance Hydrogen Evolution

Xinwu Xu[1+], Yang Lu[1+], Junqing Shi[1*], Xiaoyu Hao[1], Zelin Ma[1], Ke Yang[1], Tianyi Zhang[1], Chan Li[1], Dina Zhang[1], Xiaolei Huang[2*], Yibo He[1*]

[1] State Key Laboratory of Solidification Processing, Center of Advanced Lubrication and Seal Materials, School of Materials Science and Engineering, Northwestern Polytechnical University, Xi'an, Shaanxi 710072, P. R. China.

[2] Institute of Material and Chemistry, Ganjiang Innovation Academy, Chinese Academy of Sciences, Ganzhou, 341000, China.

*Correspondence to: junqin.shi@nwpu.edu.cn (J.S.); xlhuang@gia.cas.cn (X.H.); heyibo@nwpu.edu.cn (Y.H.)

+These authors contributed equally to this work.

Supplementary Figures

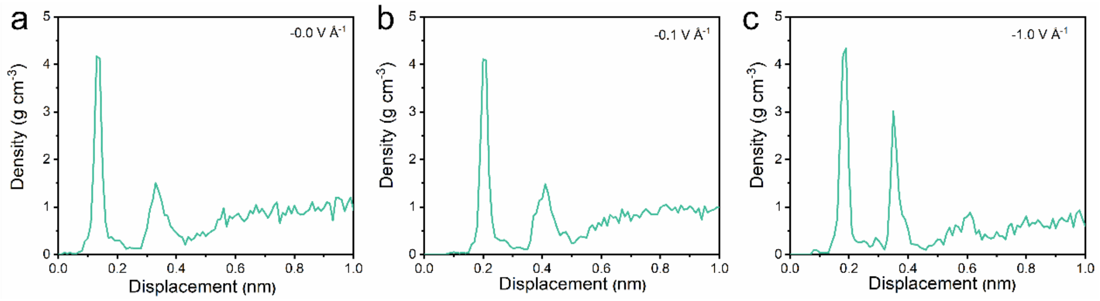

Supplementary Fig. 1 MD simulations. a-c) Mass density of H_2O molecules versus the distance above the electrode surface with the presence of static external electric fields (0, −0.1, and −1.0 V $Å^{-1}$).

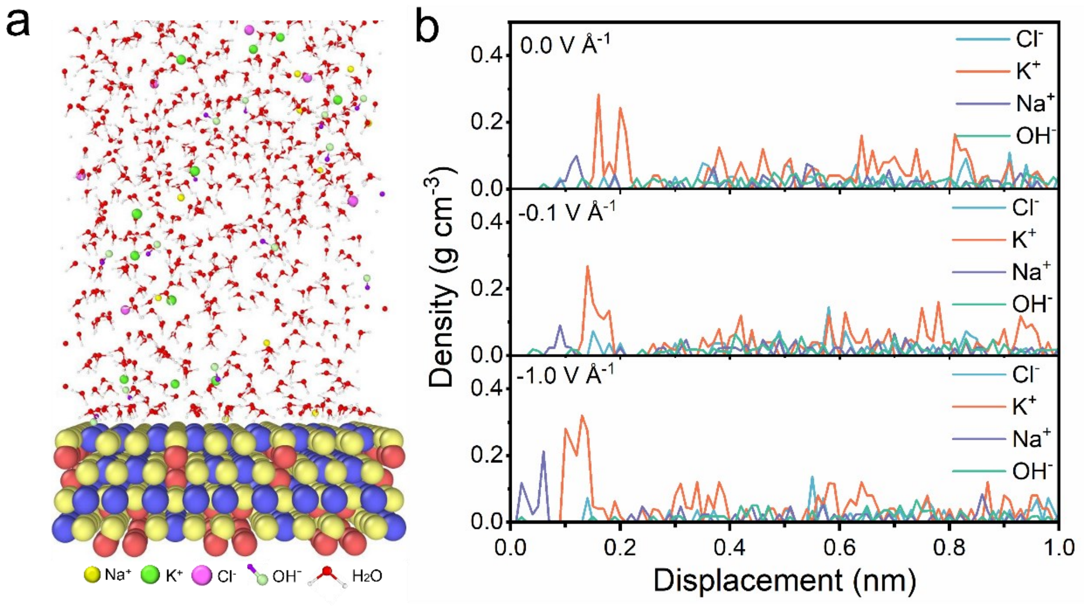

Supplementary Fig. 2 MD simulations. a) Equilibrium configuration of electrolyte system (1.0 M KOH + 0.6 M NaCl) above the electrode surface of Co_3O_4 with the presence of static external electric fields (–1.0 V $Å^{-1}$), viewed from XZ cross-section.

b) Number density of various anions versus the distance above the electrode surface with the presence of static external electric fields (0, –0.1, and -1.0 V $Å^{-1}$).

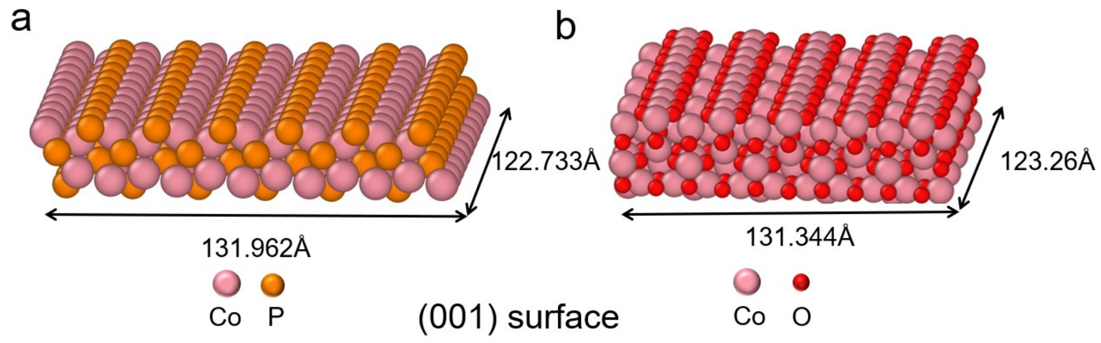

Supplementary Fig. 3 Schematic diagram of a) CoP, b) Co_3O_4 substrate.

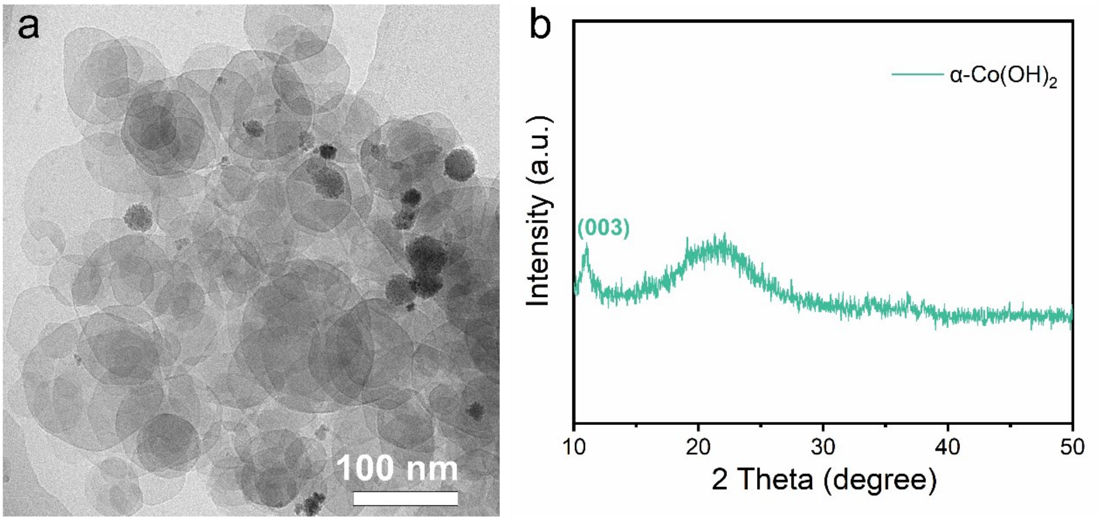

Supplementary Fig. 4 Morphology and phase characterization. a) TEM image and b) XRD pattern of α-$Co(OH)_2$.

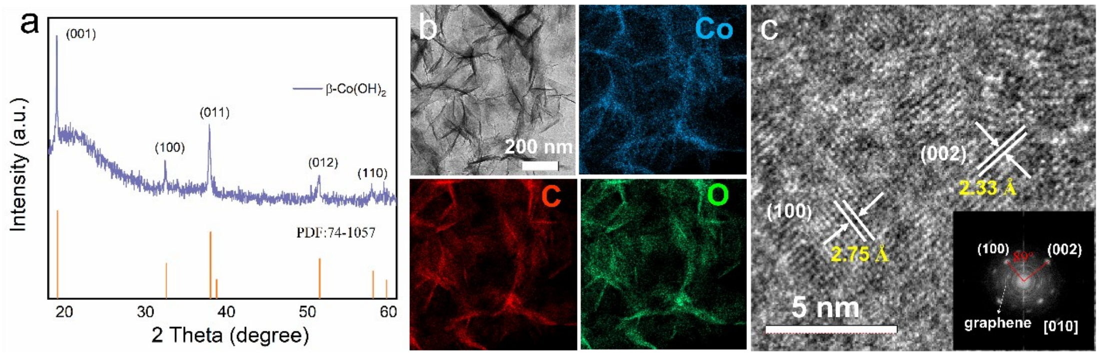

Supplementary Fig. 5 Morphology and phase characterization. a) XRD pattern and b) *β*-$Co(OH)_2$/rGO and corresponding elemental mapping images including Co, C and O elements. c) HRTEM image of *β*-$Co(OH)_2$/rGO, inset: FFT pattern of *β*-$Co(OH)_2$/rGO.

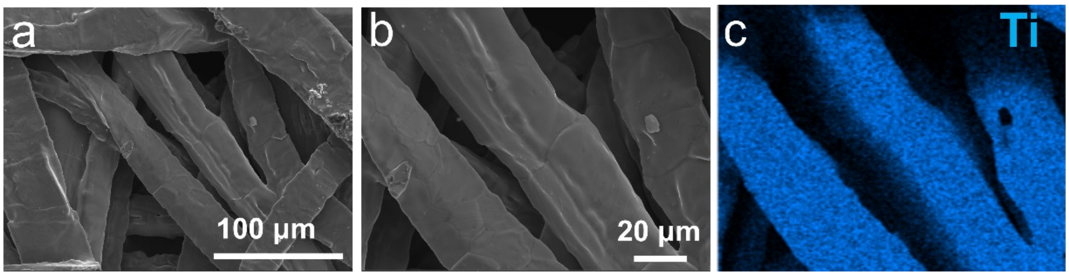

Supplementary Fig. 6 Morphology characterization. a) Low-magnification SEM images and b) high-magnification SEM images of Ti fiber felt. c) Corresponding elemental mapping image of (b).

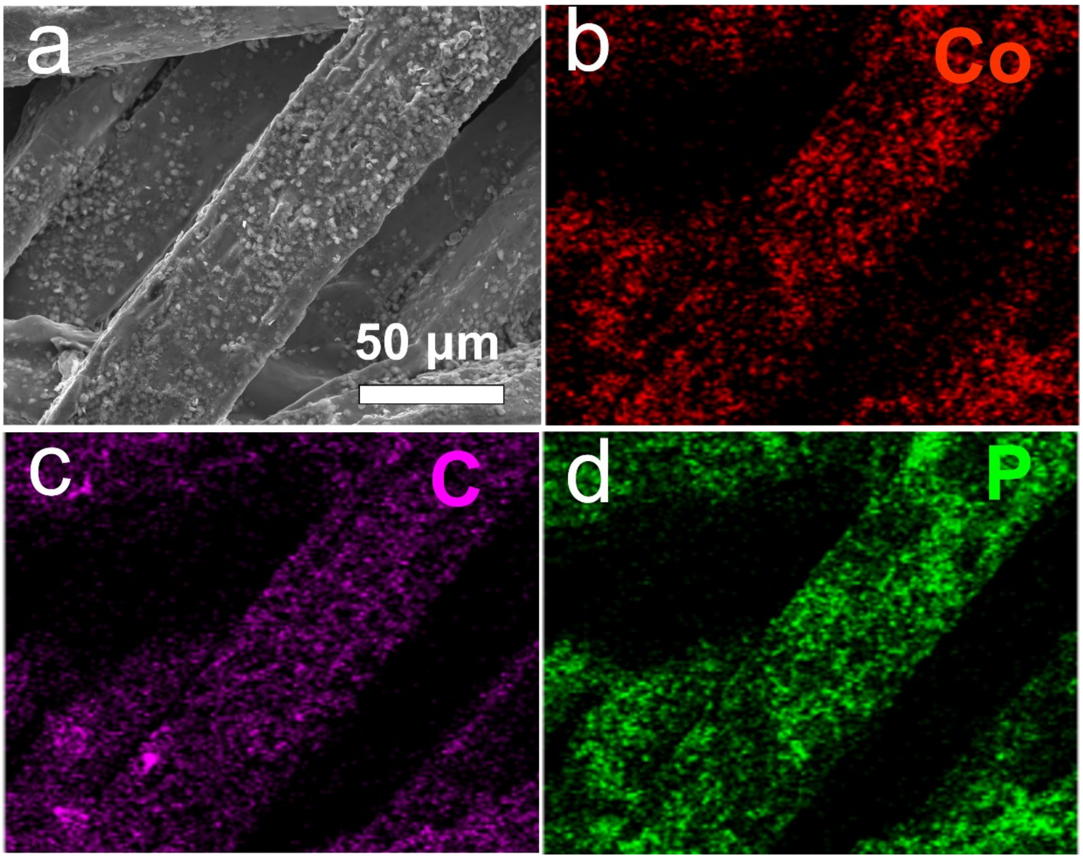

Supplementary Fig. 7 Morphology characterization. a) SEM image and the corresponding elemental mapping images b) Co, c) C, d) P elements of CoPGT.

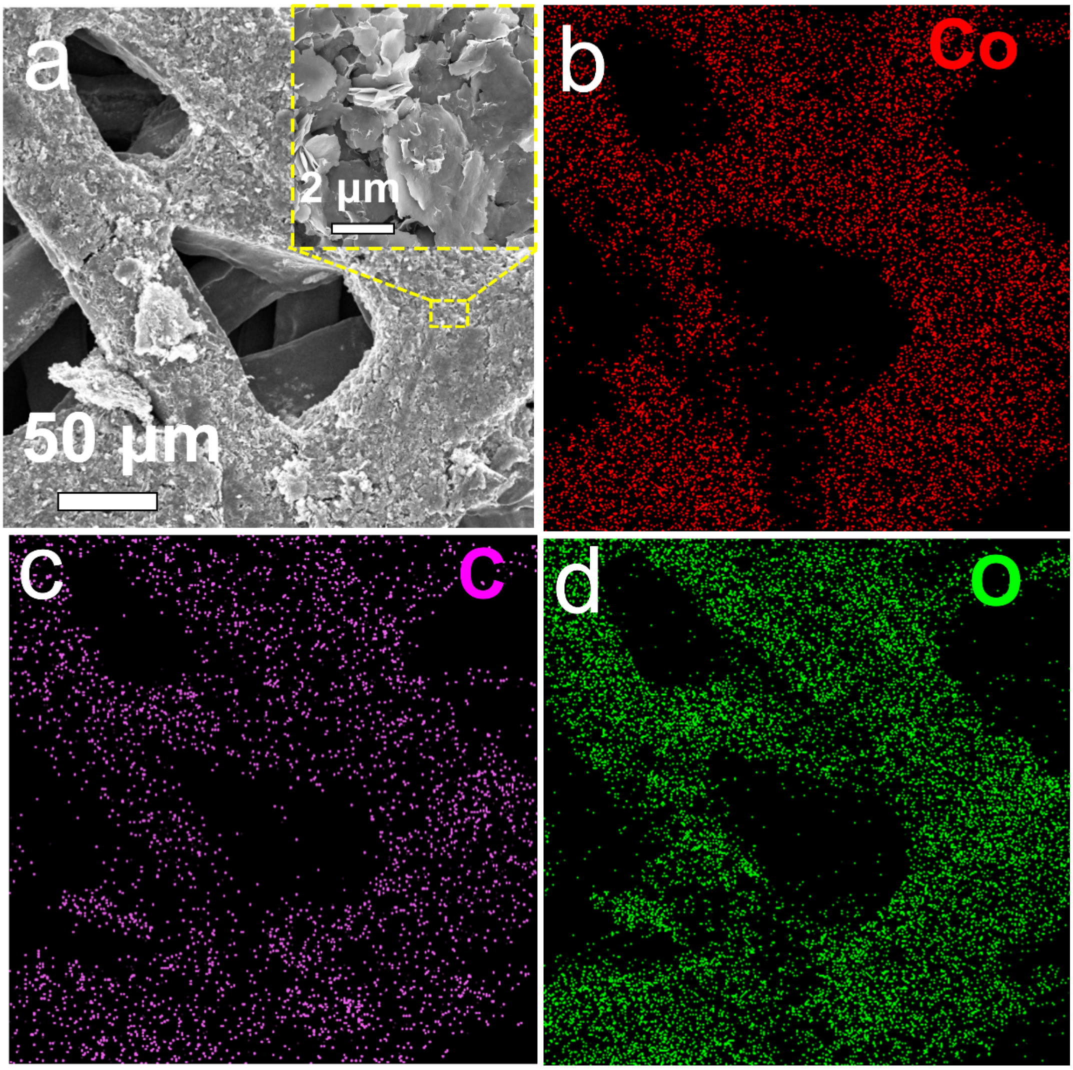

Supplementary Fig. 8 Morphology characterization. a) SEM image and the corresponding elemental mapping images b) Co, c) O, d) C elements of Co_3O_4GT.

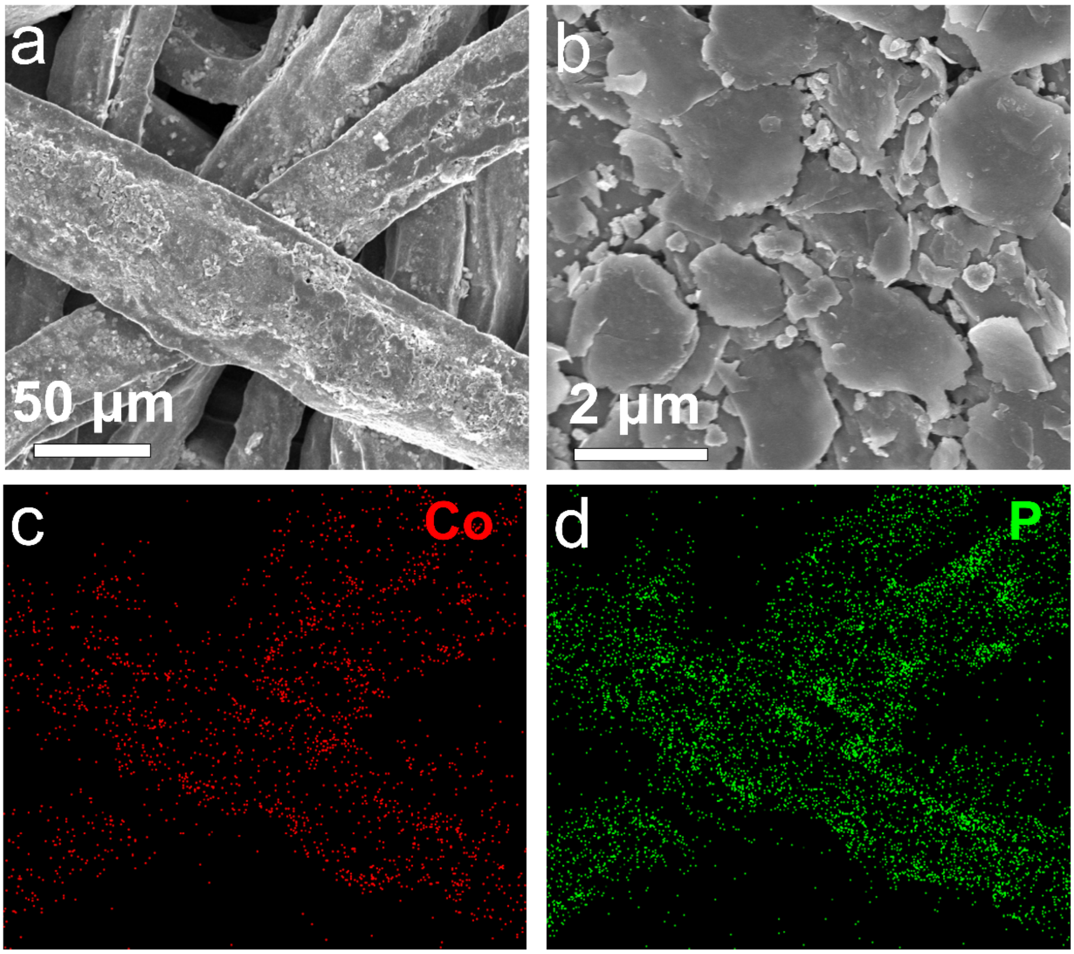

Supplementary Fig. 9 Morphology characterization. a-b) SEM image and the corresponding elemental mapping images c) Co, d) P elements of CoPT.

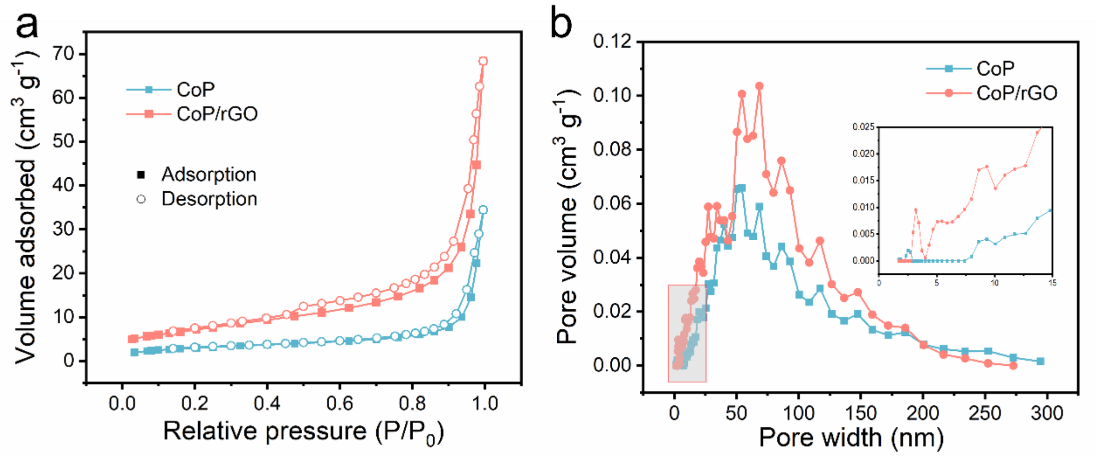

Supplementary Fig. 10 Specific surface area and pore size characterization. a) Nitrogen adsorption/desorption isotherms and b) the BJH pore-size distribution curves of CoP and CoP/rGO, respectively.

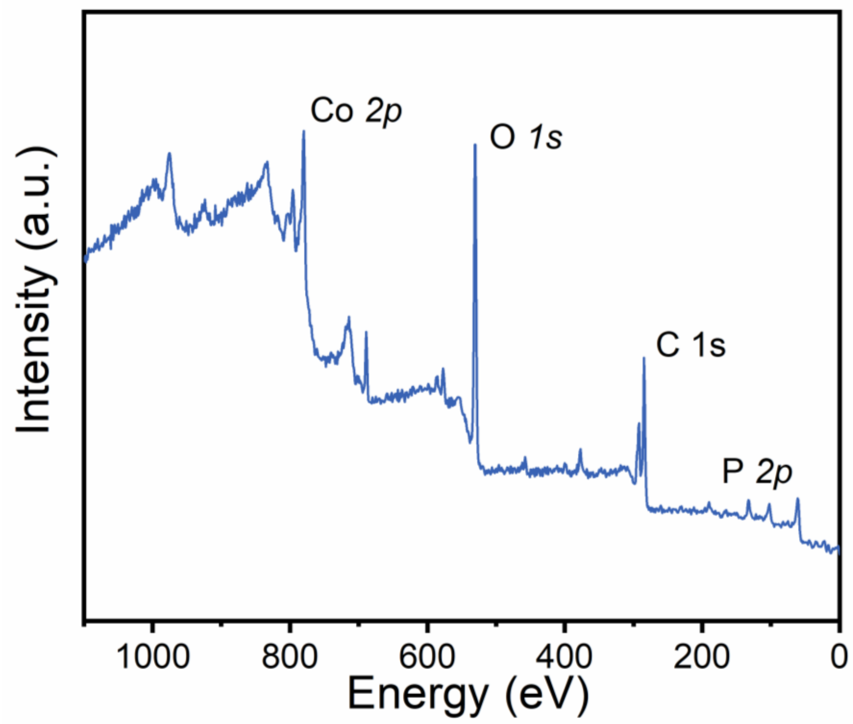

Supplementary Fig. 11 Surface elemental composition analysis. XPS survey spectra of CoPGT.

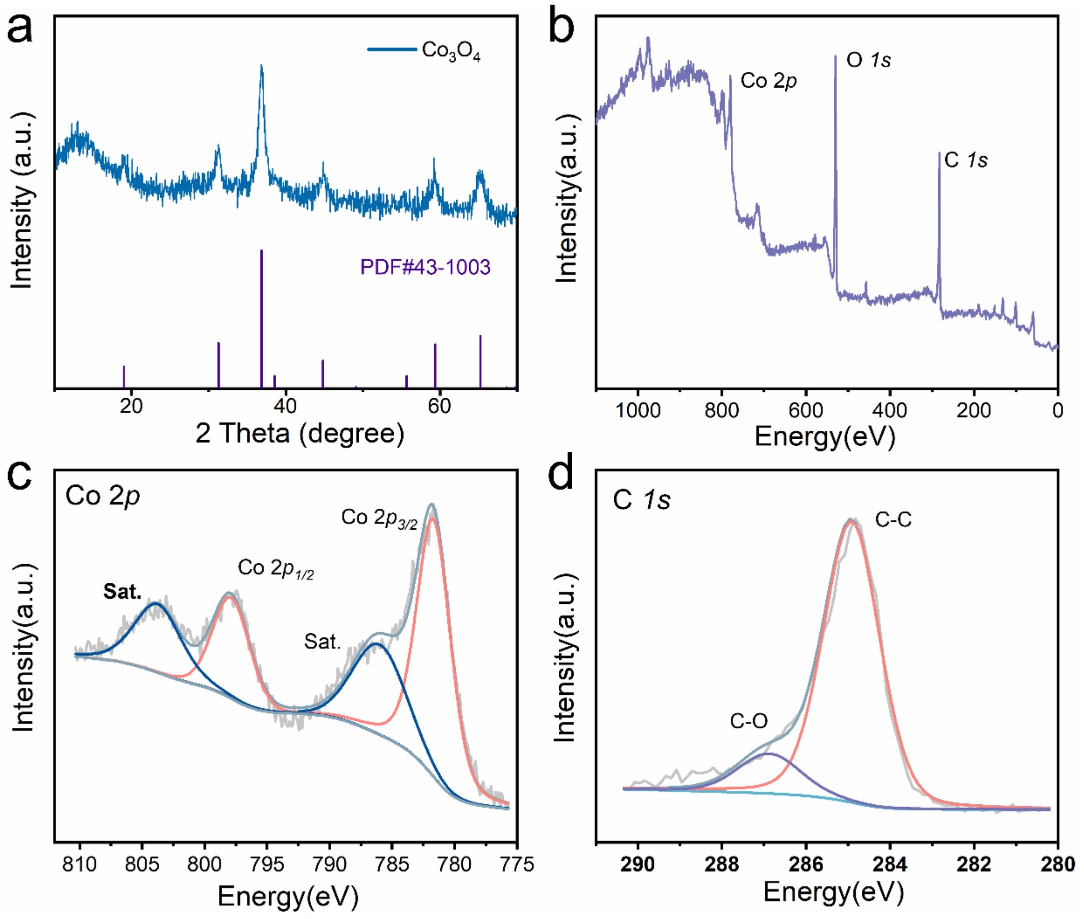

Supplementary Fig. 12 Surface elemental composition analysis. a) XRD pattern of Co_3O_4. b) XPS survey spectra of Co_3O_4GT and high-resolution c) Co*2p*, d) C*1s*. The XRD pattern of Co_3O_4 and XPS spectrums of Co_3O_4GT confirm the oxidized Co species could be attributed to Co_3O_4.

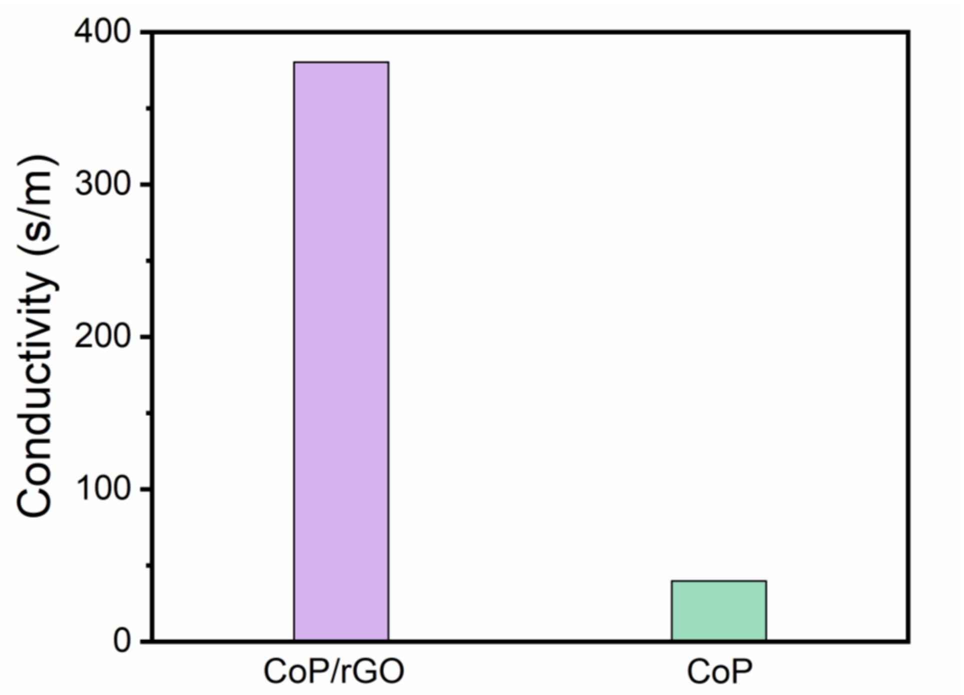

Supplementary Fig. 13 Conductivity analysis. Conductivity of CoP/rGO and CoP powder.

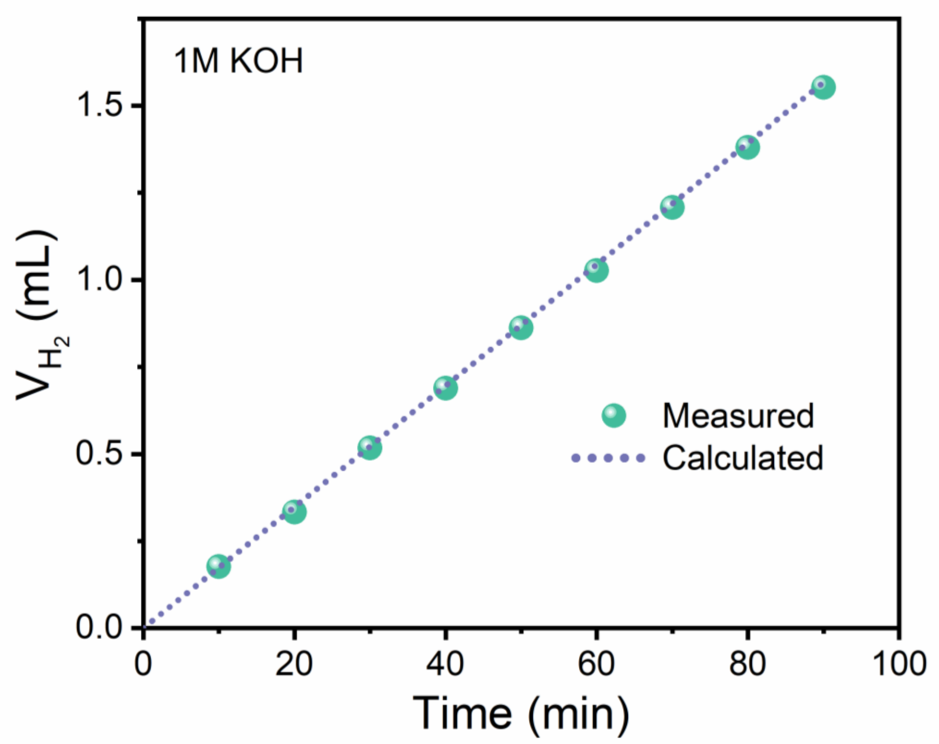

Supplementary Fig. 14 Faradic efficiency measurements. The amount of H_2 experimentally measured and theoretically calculated versus time for CoP/rGO in 1.0 M KOH.

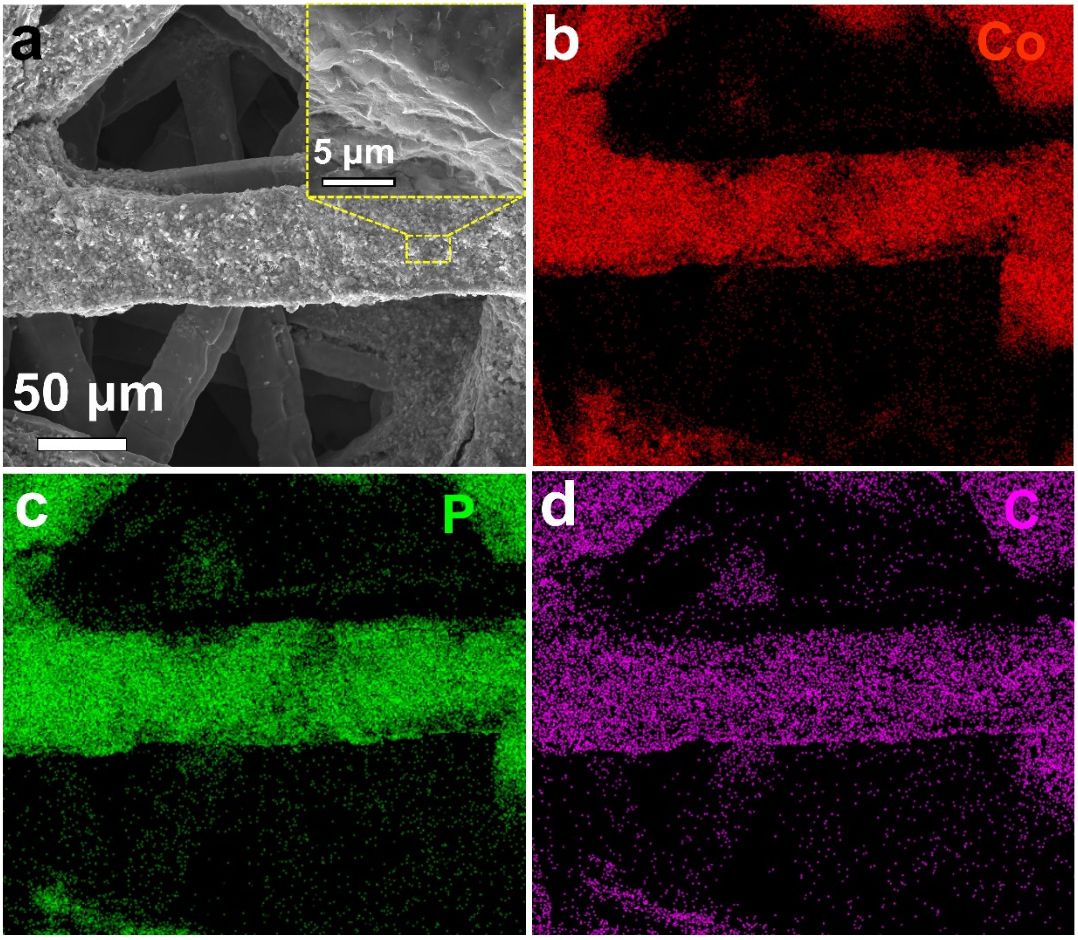

Supplementary Fig. 15 Morphology characterization. a) SEM image and corresponding elemental mapping images including b) Co, c) C and d) O elements of CoPGT after 50 h CP tests in 1.0 M KOH.

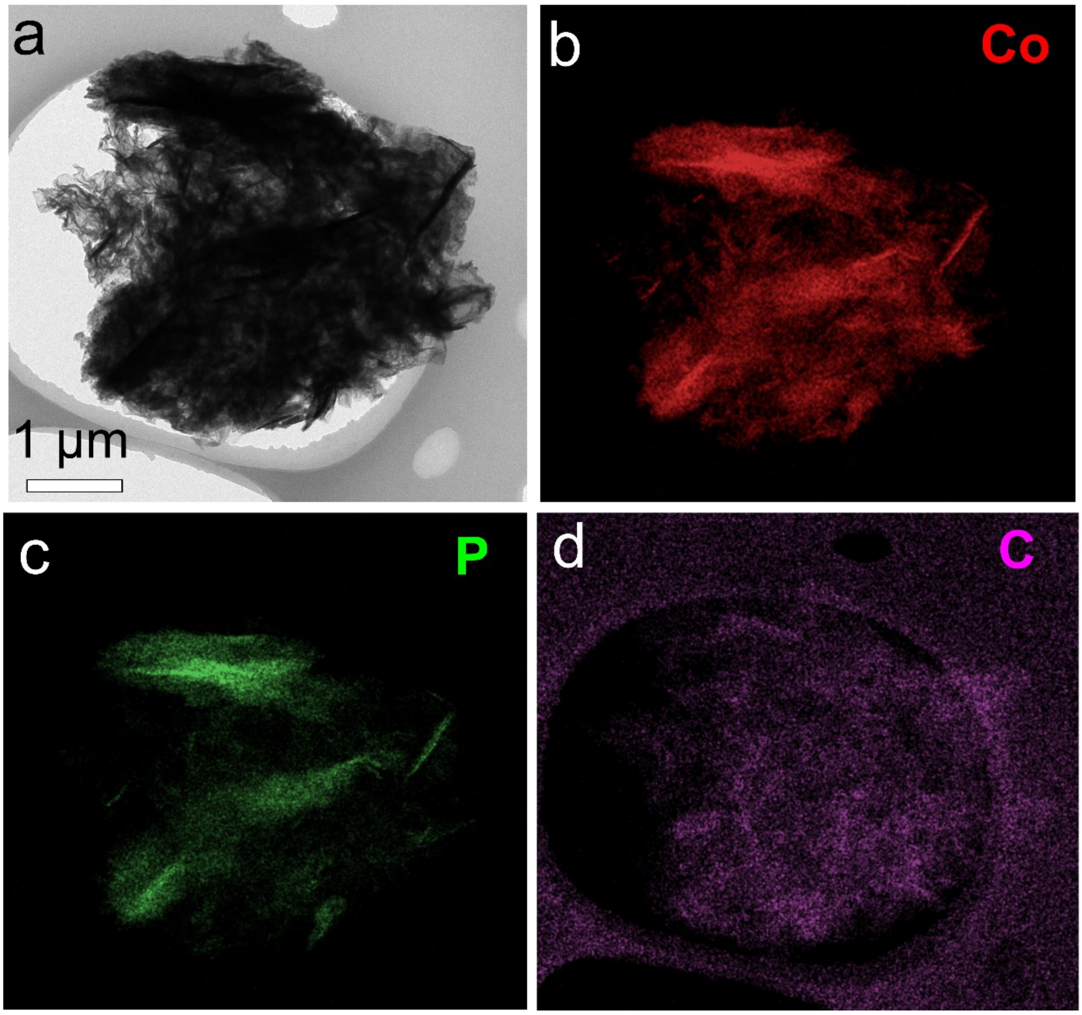

Supplementary Fig. 16 Morphology characterization. a) TEM image and the corresponding elemental mapping images b) Co, c) P, d) C elements of CoP/rGO after 50 h CP tests in 1.0 M KOH.

.

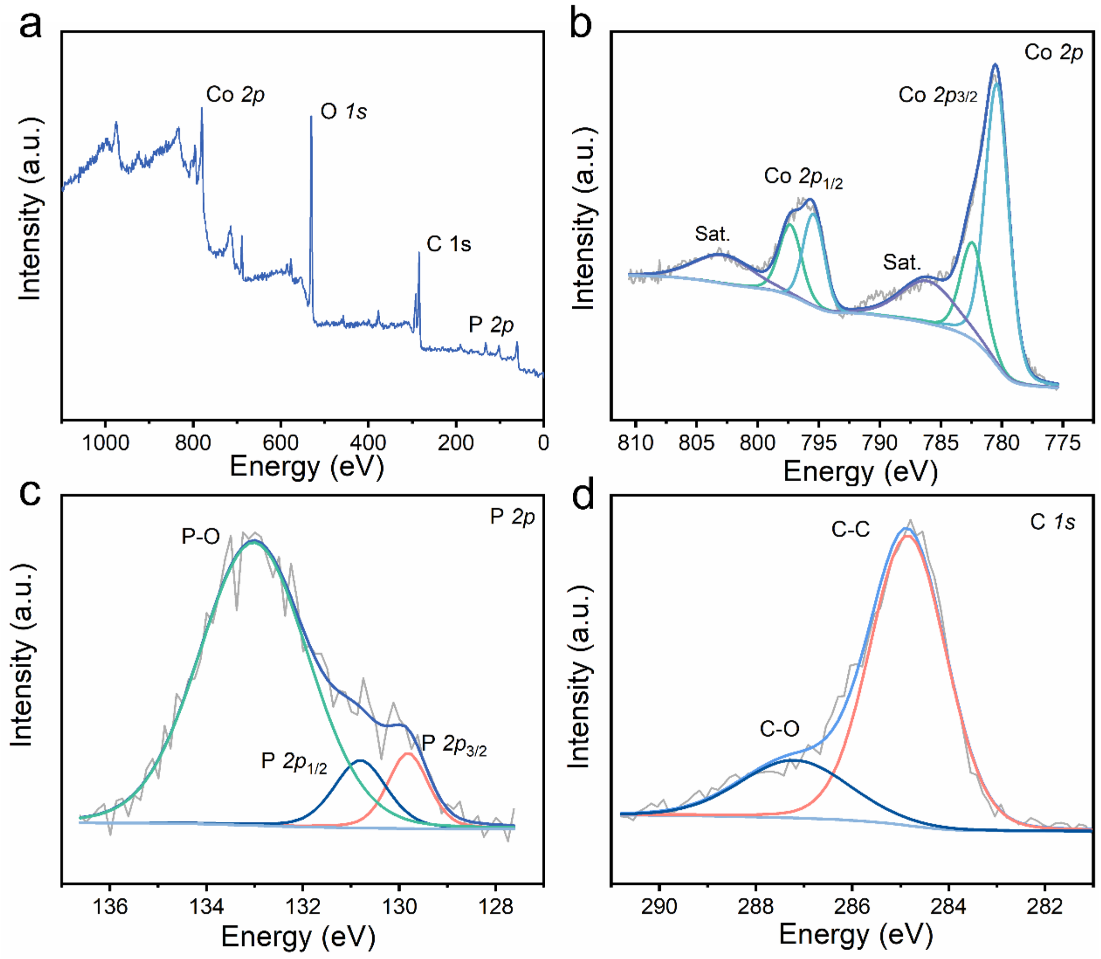

Supplementary Fig. 17 Surface elemental composition analysis. a) XPS survey spectra of CoPGT and high-resolution spectra of b) Co *2p*, c) P *2p* and d) C *1s* after 10 h CP tests in 1.0 M KOH.

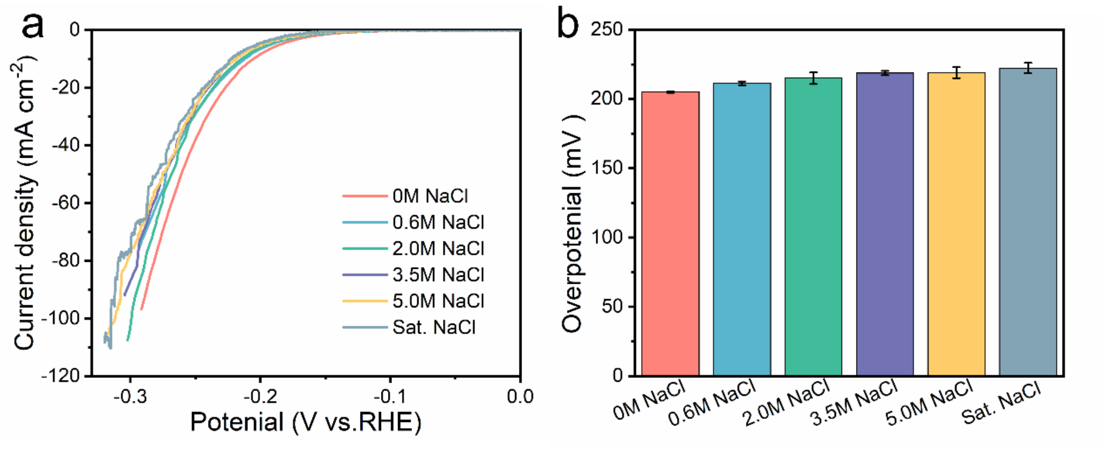

Supplementary Fig. 18 Electrochemical HER performance measurements. a) LSV curves (iR compensated) of CoP catalyst ink for the HER in different salt solutions with 1.0 M KOH. b) Corresponding variogram of overpotential at 10 mA cm^{-2}. All electrolytes with 1.0 M KOH.

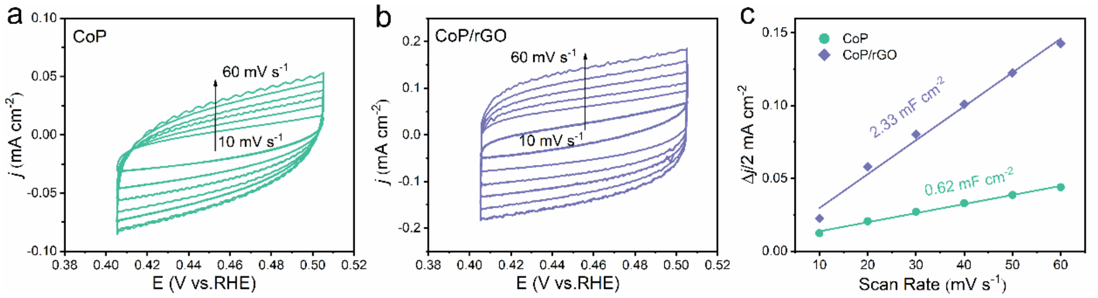

Supplementary Fig. 19 Determination of C_{dl}. CV curves of a) CoP, b) CoP/rGO with various scan rates of 10, 20, 30, 40, 50 and 60 mV s^{-1} in the non-faradaic region. c) The capacitive current densities as a function of scan rate for the catalysts.

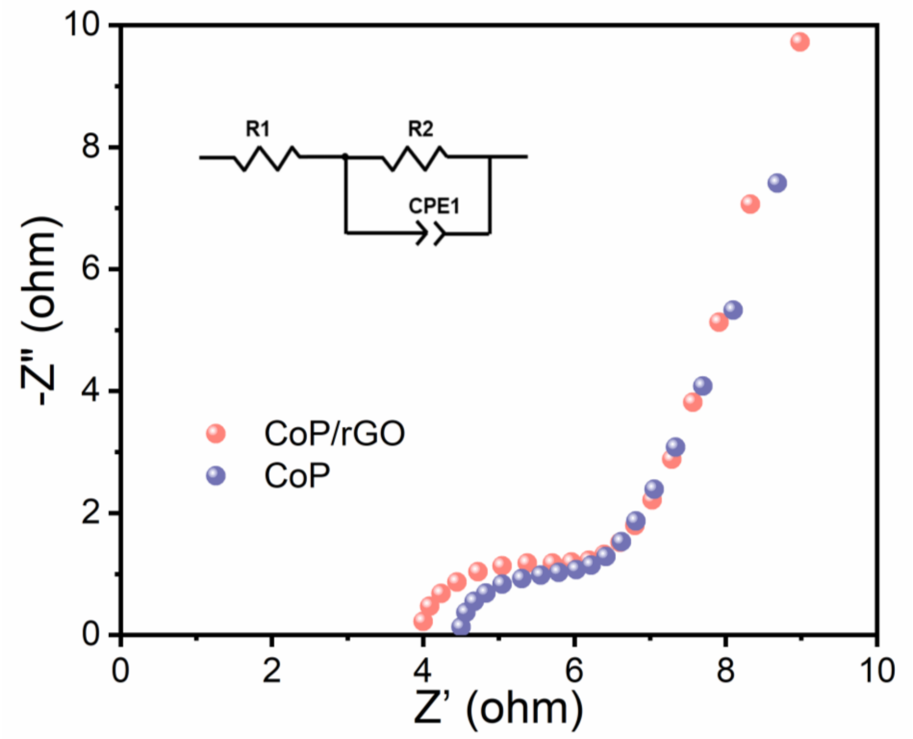

Supplementary Fig. 20 EIS Nyquist plots of CoP/rGO and rGO.

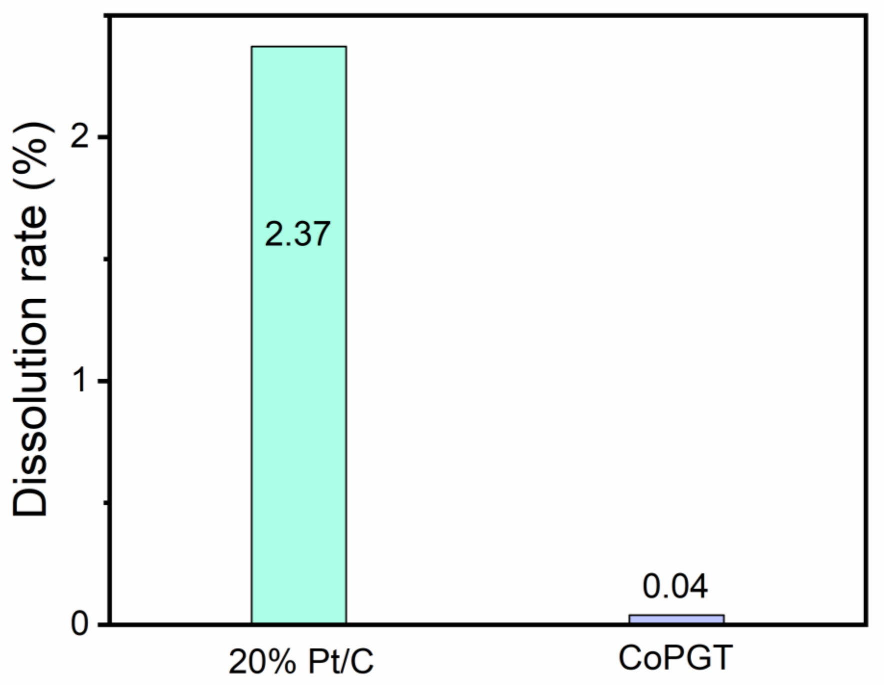

Supplementary Fig. 21 Study for the dissolution of 20% Pt/C and CoPGT in water splitting. Dissolution of 20% Pt/C and CoPGT in 0.6 M NaCl+1.0 M KOH. (The data obtained by an inductively coupled plasma mass spectrometry (ICP−MS) analysis).

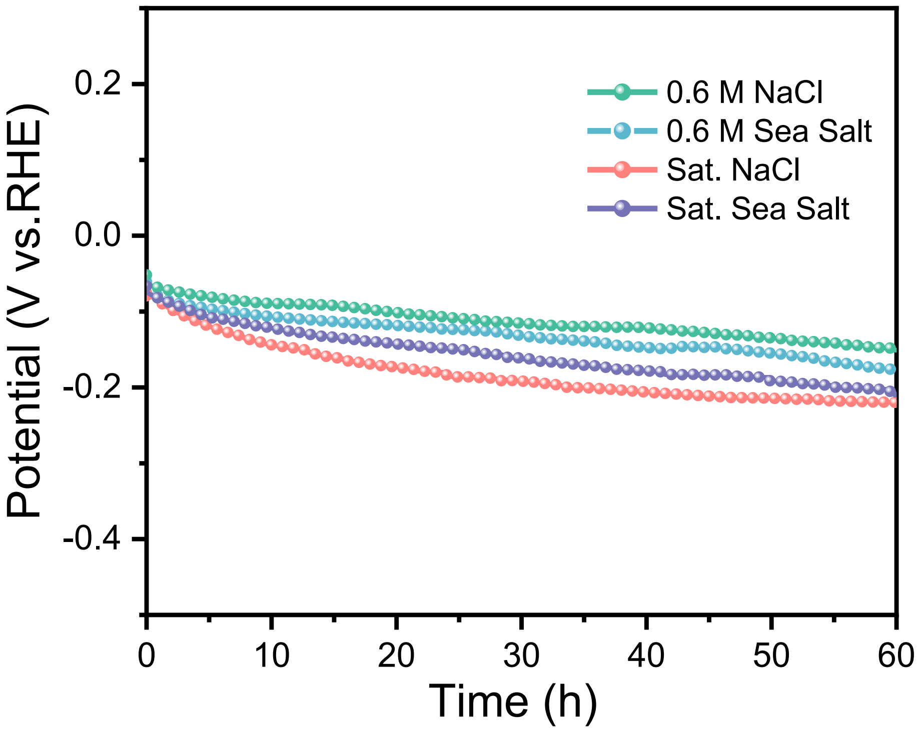

Supplementary Fig. 22 Electrochemical HER performance measurements. Chronopotentiometry curves of 20% Pt/C at 10 mA cm^{-2} in 1.0 M KOH with 0.6 M NaCl, saturated NaCl, 0.6 M sea salt and saturated sea salt, respectively.

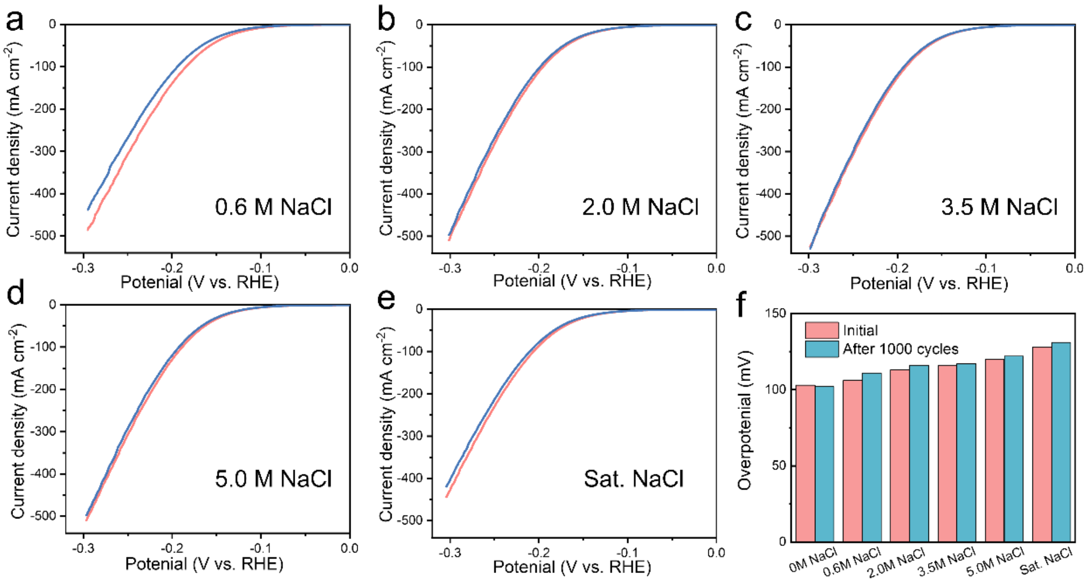

Supplementary Fig. 23 LSV cycling test. a-e) Polarization curves of CoPGT before and after 1000 LSV cycles in different concentration of NaCl electrolyte. f) The histogram of overpotential for CoPGT initially and after 1000 cycles at a scan rate of 1 mV s^{-1} for HER at 10 mA cm^{-2}. All electrolytes with 1.0 M KOH.

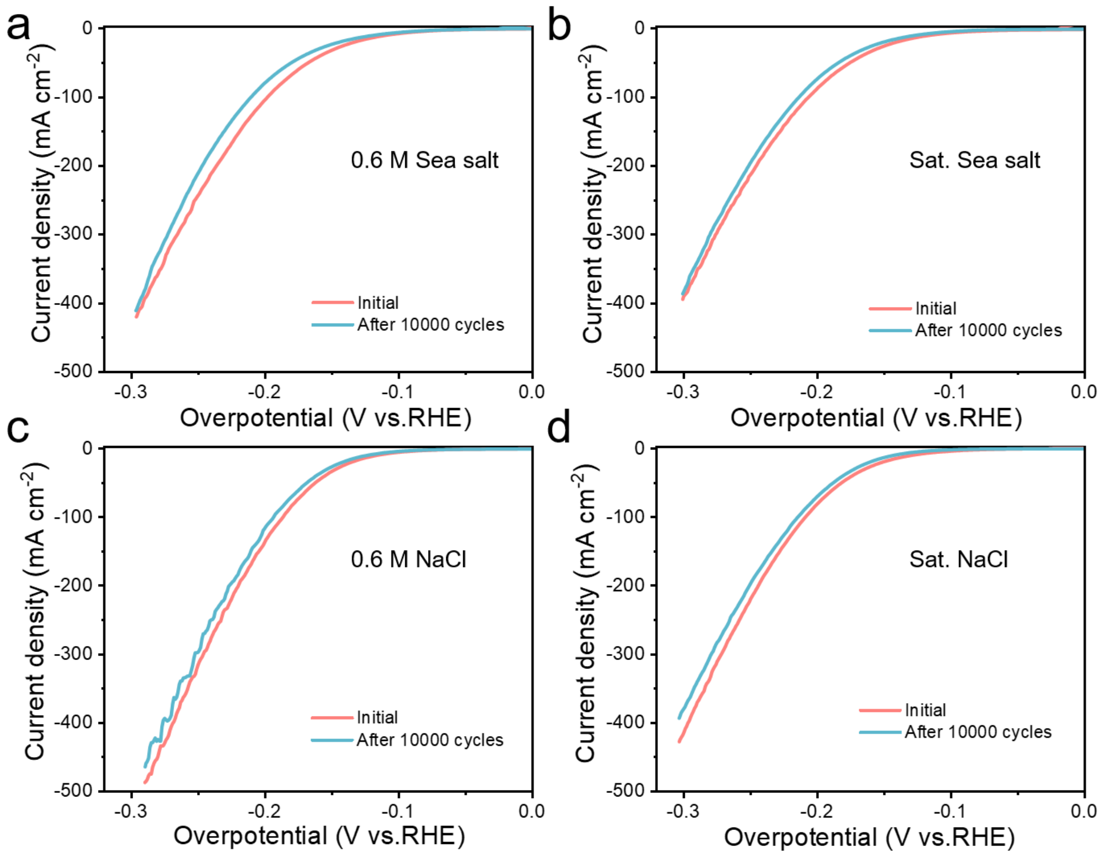

Supplementary Fig. 24 Salinity resistance performance. Polarization curves of CoPGT initially and after 10000 cycles at a scan rate of 1 mV s^{-1} for HER in 1.0 M KOH with a) 0.6 M sea salt and b) saturated sea salt, c) 0.6 M NaCl, d) saturated NaCl, respectively.

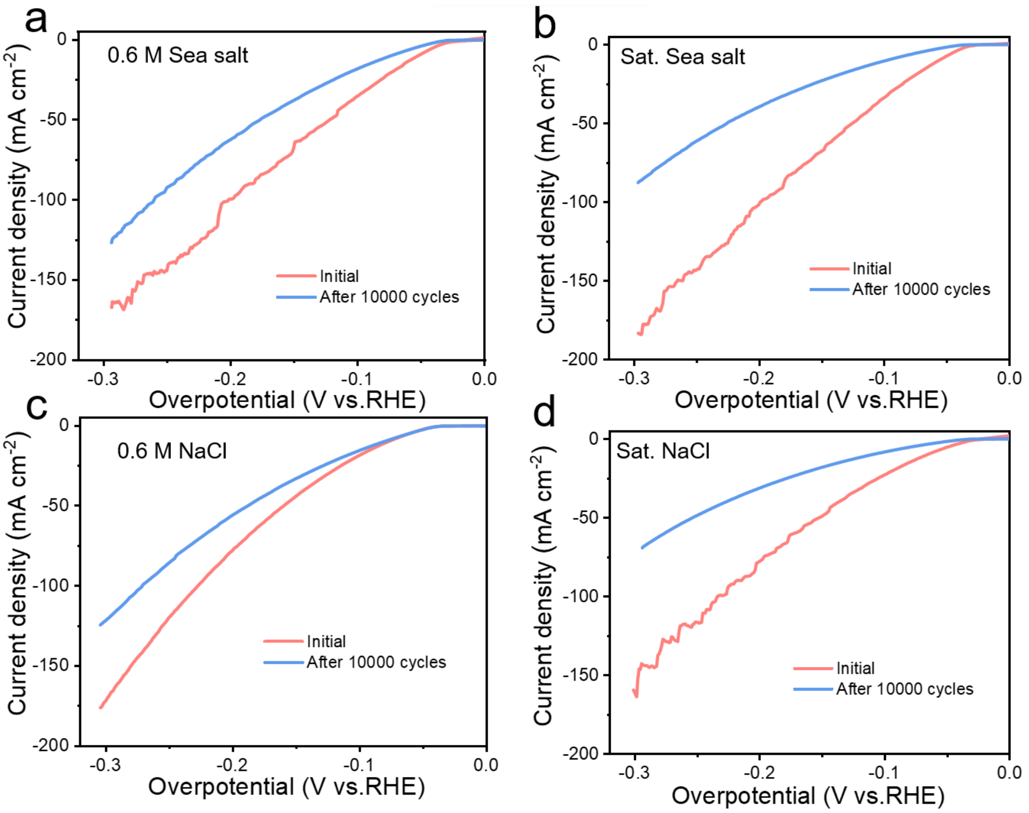

Supplementary Fig. 25 Salinity resistance performance. Polarization curves of 20% Pt/C initially and after 10000 cycles at a scan rate of 1 mV s^{-1} for HER in 1.0 M KOH with a) 0.6 M sea salt and b) saturated sea salt, c) 0.6 M NaCl, d) saturated NaCl, respectively.

Supplementary Table 1 LJ parameters for the saline ions, H_2O with substrates.

\		ε (kcal/mol)	σ (Å)	q (e)
Na^+		0.3526418[1]	2.1595384928[1]	+1
Cl^-		0.0127850[1]	4.8304528498[1]	-1
K^+		0.4297054[1]	2.838403315[1]	+1
OH^-	O	0.2280000124[3]	2.8597848722[3]	-1
	H	0[3]	0[3]	0
H_2O	H	0[2]	0[2]	0.4238
	H	0[2]	0[2]	0.4238
	O	0.1553[2]	3.166[2]	-0.8476
CoP	Co	0.0286[4]	1.2267[4]	2.43
	P	0.1999976833[3]	3.7417782334[3]	-2.43
Co_3O_4	Co	0.0286[4]	1.2267[4]	2.43
	O	0.2280000124[3]	2.8597848722[3]	-1.8225

Supplementary Table 2 Comparison of the electrocatalytic HER properties of recently reported HER electrocatalysts in alkaline seawater.

Catalyst	Current density (j/mA cm^{-2})	Overpotential at the corresponding j (mV)	Tafel slope (mV dec^{-1})	Ref.
S,P-(Ni,Mo,Fe) OOH/NiMoP/wood	50	187	1 M KOH + seawater	[5]
FeNiP-NPHC	100	180	1 M KOH +seawater	[6]
Co-Fe_2P	10	85	simulated alkaline seawater	[7]
NiMoN@NiFeN	100	82	1 M KOH + seawater	[8]
Cu_2S	10	118	1 M KOH + seawater	[9]
Er-MoO_2	10	173	1.0 M KOH + 0.5 M NaCl	[10]
Ru, W–$NiSe_2$/NF	10	353	Seawater	[11]
Ni-WO_x@NF	10	46	1 M KOH + Seawater	[12]
$CoNiSe_2$/ N-SSCSs	10	105	alkaline simulated seawater	[13]
PF-NiCoP/NF	10	287	seawater	[14]
NiFeS/NF	100	217	1 M KOH + Seawater	[15]
CoPx@FeOOH	10	117	1 M KOH + seawater	[16]
CoP/Co_2P	10	454	simulated seawater	[17]
Co-Ni-P/CP	10	108	Simulated alkaline seawater	[18]
$NiRu_{0.13}$-BDC	10	36	1 M phosphate buffered saline solution	[19]
Cr-Co_XP	100	194	1 M KOH + seawater	[20]
Ni-SA/NC	10	139	1 M KOH + seawater	[21]
Ni_2P-Fe_2P/NF	10<br>100	220<br>252	1 M KOH + seawater	[22]
CoPGT	10<br>100	110<br>192	1 M KOH + 0.6 M NaCl	This work

Supplementary References

1. Nagashima, S. et al. Atomic-Level Observation of Electrochemical Platinum Dissolution and Redeposition. *Nano Lett.* **19**, 7000–7005 (2019).
2. Joung, I. S. & Cheatham, T. E. I. Determination of Alkali and Halide Monovalent Ion Parameters for Use in Explicitly Solvated Biomolecular Simulations. *J. Phys. Chem. B* **112**, 9020–9041 (2008).
3. Berendsen, H. J. C., Grigera, J. R. & Straatsma, T. P. The missing term in effective pair potentials. *J. Phys. Chem.* **91**, 6269–6271 (1987).
4. Maple, J. R., Dinur, U. & Hagler, A. T. Derivation of force fields for molecular mechanics and dynamics from ab initio energy surfaces. *Proc. Natl. Acad. Sci.* **85**, 5350–5354 (1988).
5. Chen, H. *et al.* Wood aerogel-derived sandwich-like layered nanoelectrodes for alkaline overall seawater electrosplitting. *Appl. Catal. B: Environ.* **293**, 120215 (2021).
6. Yu, Q. *et al.* Constructing three-phase heterojunction with 1D/3D hierarchical structure as efficient trifunctional electrocatalyst in alkaline seawater. *Adv. Funct. Mater.* **32**, 2205767 (2022).
7. Wang, S. *et al.* Synthesis of 3D heterostructure Co-doped Fe_2P electrocatalyst for overall seawater electrolysis. *Appl. Catal. B: Environ.* **297**, 120386 (2021).
8. Yu, L. *et al.* Non-noble metal-nitride based electrocatalysts for high-performance alkaline seawater electrolysis. *Nat. Commun.* **10**, 5106 (2019).
9. Marimuthu, T. *et al.* Cost effective and facile low temperature hydrothermal fabrication of Cu2S thin films for hydrogen evolution reaction in seawater splitting. *Int. J. of Hydrogen Energy* **47**, 30819–30829 (2022).
10. Yang, T. *et al.* Electronic structure modulation of MoO_2 via Er-doping for efficient overall water/seawater splitting and Mg/seawater batteries. *Appl. Surf. Sci.* **615**, 156360 (2023).
11. Dang, Y. *et al.* Enhanced alkaline/seawater hydrogen evolution reaction performance of $NiSe_2$ by ruthenium and tungsten bimetal doping. *Int. J. of Hydrogen Energy* (2023).
12. Liang, W. *et al.* Nickel-doped tungsten oxide promotes stable and efficient hydrogen evolution in seawater. *Appl. Catal. B: Environ.* **325**, 122397 (2023).
13. Wang, Z. *et al.* Integrating $CoNiSe_2$ nanorod-arrays onto N-doped sea-sponge-C spheres

for highly efficient electrocatalysis of hydrogen evolution reaction. *Chem. Eng. J.* **446**, 137335 (2022).

14. Lv, Q. *et al.* Featherlike NiCoP holey nanoarrys for efficient and stable seawater splitting. *ACS Appl. Energ. Mater.* **2**, 3910-+ (2019).
15. Chen, J. *et al.* High-efficiency overall alkaline seawater splitting: using a nickel–iron sulfide nanosheet array as a bifunctional electrocatalyst. *J. Mater. Chem. A* **11**, 1116–1122 (2023).
16. Wu, L. *et al.* Rational design of core-shell-structured CoPx@FeOOH for efficient seawater electrolysis. *Appl. Catal. B: Environ.* **294**, 120256 (2021).
17. Zhang, J. *et al.* Surface-electronic-structure reconstruction of perovskite via double-cation gradient etching for superior water oxidation. *Nano Lett.* **21**, 8166–8174 (2021).
18. Yu, Z., Xu, J., Meng, L. & Liu, L. Efficient hydrogen production by saline water electrolysis at high current densities without the interfering chlorine evolution. *J. Mater. Chem. A* **9**, 22248–22253 (2021).
19. Sun, Y. *et al.* Modulating electronic structure of metal-organic frameworks by introducing atomically dispersed Ru for efficient hydrogen evolution. *Nat. Commun.* **12**, 1369 (2021).
20. Song, Y. *et al.* Alleviating the work function of vein-like Co_XP by Cr doping for enhanced seawater electrolysis. *Adv. Funct. Mater.*, 2214081.
21. Zang, W. *et al.* Efficient hydrogen evolution of oxidized Ni-N_3 defective sites for alkaline freshwater and seawater electrolysis. *Adv. Mater.* **33**, 2003846 (2021).
22. Wu, L. *et al.* Heterogeneous bimetallic phosphide Ni_2P-Fe_2P as an efficient bifunctional catalyst for water/seawater splitting. *Adv. Funct. Mater.* **31**, 2006484 (2021).
